# Development and psychometric testing of the patient participation in bedside handover survey

**DOI:** 10.1111/hex.13569

**Published:** 2022-07-27

**Authors:** Georgia Tobiano, Andrea P. Marshall, Therese Gardiner, Kim Jenkinson, Margaret Shapiro, Michael Ireland

**Affiliations:** ^1^ Gold Coast University Hospital Gold Coast Health Southport Queensland Australia; ^2^ NHMRC CRE in Wiser Wound Care, Menzies Health Institute Queensland Griffith University Brisbane Queensland Australia; ^3^ School of Nursing and Midwifery Griffith University Brisbane Queensland Australia; ^4^ School of Psychology and Wellbeing University of Southern Queensland Toowoomba Queensland Australia

**Keywords:** bedside handover, nurse–patient relations, patient‐centred care, patient handoff, patient participation, psychometrics, surveys and questionnaires

## Abstract

**Introduction:**

When handover is conducted at the patient's bedside, active patient participation can be encouraged, which may improve the safety and quality of care. There is a need for valid and reliable tools to measure patient perceptions of participation in bedside handover, to ensure the rising number of implementation and improvement efforts are consistently and effectively evaluated. The aim of this study is to systematically develop and evaluate the psychometric properties of a self‐report survey to measure patients' perceptions of participation in bedside handover.

**Methods:**

In Phase 1, our team developed a conceptual framework and item pool (*n* = 130). In Phase 2, content validity was assessed with four health consumers, four nurses and four researchers. Next, 10 current hospital inpatients tested the survey for end‐user satisfaction. In Phase 3, 326 inpatients completed the survey, allowing exploratory factor analysis, reliability analyses and convergent/divergent validity analyses to occur.

**Results:**

Phase 1 and 2 resulted in a 42‐item survey. In Phase 3, 321 surveys were available for analysis. Exploratory factor analysis revealed a three‐factor solution, with 24 items, which matched our conceptual framework. The three factors were: ‘Conditions for patient participation in bedside handover’, ‘Level of patient participation in bedside handover’ and ‘Evaluation of patient participation in bedside handover’. There was strong evidence for factor reliability and validity. Additionally, the correlation between factors was strong.

**Conclusion:**

This study furthers our conceptual understanding by showing that nurse facilitating behaviours are a strong precursor for patient participation and perceived handover outcomes, justifying the need for nursing training. A robust survey has been developed to measure patient perceptions of participation in bedside handover, which can effectively evaluate this approach to care. Engaging consumers and nurses as research team members was invaluable in ensuring that the survey is acceptable for end‐users.

**Patient or Public Contribution:**

A health consumer and nurse partnered as members of the research team from study inception to dissemination.

## 1 INTRODUCTION

Medical errors are now the third leading cause of death in the United States.^1^ Root cause analyses suggest that over 60% of hospital errors are due to poor communication.^2^ A critically vulnerable point in the administration of inpatient healthcare is nurse shift‐to‐shift handover in which the transfer of care and responsibility for a patient is communicated from one nurse to another.^3^ The accuracy of information exchanged at handover is an important determinant of safety and quality outcomes. Evidence shows bedside handover, when accomplished well, can decrease patient falls, improve discharge times, reduce overtime costs and enhance team collaboration.^4^, ^5^ Internationally, patient participation in care is advocated as an essential strategy to improve the safety and quality of care.^6^ The occurrence of shift‐to‐shift handover at the patient's bedside represents a powerful juncture at which to actualize patient participation. However, enabling patient participation in activities like shift‐to‐shift handover can be challenging.

### 1.1 Background

Bedside handover affords a predictable and practical opportunity to meaningfully involve patients in their care. There are various ways that patients can participate in bedside handover, such as listening to, adding, clarifying, asking or answering questions and/or identifying missed information.^7^ Importantly, when patients participate in bedside handover, they are more satisfied, better informed and can improve their own safety by identifying errors in information transferred.^4^, ^5^ Ultimately, when bedside handover is optimally performed, a patient‐centred approach is enacted,^8^ and patients reap the benefits of this approach to care. Despite these benefits, international data suggest that patient participation in bedside handover occurs in as few as 5% of handovers.^3^ Researchers have concluded that patient preferences for participation in bedside handover do vary between patients.^9^ In a recent Australian study, 25% of patients were active participants, 35% passive participants and 40% had no role in shift‐to‐shift handover (passive participants included patients who had handover conducted away from their bedside).^9^ Interestingly, a recent publication found that not a single patient refused handover at the bedside, suggesting patient resistance is not a driver of the low rates of patient participation.^10^ A contributing factor may be patient confidence; when patients feel well‐equipped to voice concerns and take responsibility for their care, they are more likely to actively contribute to care.^11^ Thus, understanding patient factors may be critical to realizing the benefits of patient participation.^7^

Invariably, nurses determine handover location without consultation with the patient.^10^ Researchers suggest that 71.5%–76.9% of handovers occur at the bedside, representing many missed opportunities for patient engagement.^9^, ^10^ For this and other reasons, nurse behaviour is key to the occurrence and success of bedside handover. Nurses displaying controlling and uninviting behaviours towards patients (i.e., turning their backs towards patients)^12^ make patients feel precluded or ignored during handover.^9^ While nurse behaviours like asking patients questions and making eye contact with the patient are significantly associated with more active patient participation.^9^ Therefore, nurse facilitation is a central component of patient participation and likely an important determinant of positive outcomes.

Publications about implementing, improving and sustaining bedside handover are sharply increasing.^7^ Most previous researchers wanting to improve, implement or evaluate bedside handover have done so within a quality improvement paradigm, using evaluative surveys without evidence for their validity or reliability.^7^ In the current study, we used a rigorous test construction and validation approach to develop a tool to measure patients' perceptions of participation in bedside handover. The instrument specifically attempts to contravariance in aspects relating to the conditions, processes and outcomes of patient participation in bedside handover. This tool can assist in evaluating views of patient participation to ensure future implementation/improvement/sustainment initiatives are validity appraised.

### 1.2 Study aims

The aim of this study was to systematically develop and psychometrically evaluate a self‐report survey to measure patients' perceptions of participation in bedside handover. To achieve our aim, we: (1) inductively and deductively generated a conceptual framework to underpin survey development and an item pool; (2) pilot‐tested the survey for end‐user satisfaction and evaluated content validity and (3) fully evaluated the survey by undertaking exploratory factor analysis (EFA), reliability analyses and convergent/divergent validity analyses.

## 2 METHODS

### 2.1 Study design

In Phase 1, a conceptual framework was developed based on theory and our systematic review. Next, items were developed based on our conceptual framework, existing measures, our systematic review and previous qualitative and quantitative work. Phase 2 followed an exploratory cross‐sectional approach involving pilot testing of the item pool for content validity and end‐user satisfaction. Phase 3 was an evaluative cross‐sectional design adopting a correlational analytic approach to evaluate the psychometric quality of the instrument. All phases of the research had human research ethics approval from Gold Coast Hospital and Health Service (HREC/2019/QGC/50604) and Griffith University (2019/414). All participants were provided verbal and written ethics‐approved information and return of survey implied consent.

### 2.2 Phase 1

#### 2.2.1 Concept development

Our team developed a conceptual framework based on theory and empirical literature to guide item development. Nurse–patient communication conceptual models by Fleischer et al.^13^ and Evans^14^ and our systematic review on patient participation in bedside handover^7^ collectively guided our conceptual framework (see Supporting Information: File S1 for more information), which contained the following three constructs:

1. *Conditions for patient participation in bedside handover*: A state where both patients and nurses have met the conditions for active patient participation in bedside handover. Nurse conditions include their interpersonal style and behaviours that encourage patient participation and their information‐sharing behaviours that need to be adapted to the individual. Patient conditions include their individual characteristics such as their capacity to participate in bedside handover.
2. *Level of patient participation in bedside handover*: An evaluation of levels of patient behaviour and preferences. For behaviour, it is the extent of participation a patient undertakes in bedside handover. Patient participation includes nurse–patient communication about health information, and the levels range from passive to active patient participation. Health information includes topics like patient symptoms, capabilities or usual regimens, and patients may communicate by sharing information and asking questions. Patients may also participate nonverbally, for example by listening to nurse communication. For preference, it is an assessment of their preferred levels of participation.
3. *Evaluation of patient participation in bedside handover*: An assessment of whether the level of participation a patient experienced was what they desired and resulted in positive outcomes like perceived quality of the interaction, the nurse–patient relationship, adaptation to the individual, patient understanding and patient satisfaction.

#### 2.2.2 Item development

An initial list of 130 items was generated that were believed to appropriately map the conceptual framework we developed. Two surveys were found related to patient participation in bedside handover, which were developed in a robust manner.^15^, ^16^ However, based on the conceptual framework guiding our survey development, our team identified that these surveys did not fully cover our underlying constructs. Nevertheless, with permission from the creators, items from these surveys were included in our initial pool. The development of survey items was also informed by our systematic review on patient participation in bedside handover,^7^ our interviews with patients about participation in bedside handover,^17^ a discrete choice experiment survey on patient participation in bedside handover,^18^ and previous surveys used to measure patients' perceptions of bedside handover in quality improvement projects or surveys without psychometric testing.^19^, ^20^, ^21^, ^22^, ^23^, ^24^ Our research team consisting of a consumer, a nurse, a psychometric expert and researchers reviewed and reduced the list of 130 items to 60.

### 2.3 Phase 2

Next, content experts established content validity over two rounds. The content expertize of consumers, nurses and researchers was sought due to their experience, practice or research relating to bedside handover. In Round 1, four consumers, four nurses and four researchers participated, and in Round 2, one consumer, one nurse and one researcher from Round 1 participated. Eighteen items were removed based on Polit and Beck's^25^ content validity index, which was measured at the item and scale level, and expert comments. Full details of the content validity process are in Supporting Information: File S2. In summary, over two rounds 60 items were reduced to 42 items.

Next, the list of items was formatted into a survey. We followed Pett et al.'s^26^ recommendations for font size, layout and instruction development. Further, a designer was hired to format the survey so that it was attractive and appealing to the participants and was consistent with the hospital branding. At this point, the survey utilized a 4‐point Likert‐type response scale (1 = *strongly disagree*, 2 = *disagree*, 3 = *agree* and 4 = *strongly agree*).

The survey was tested with current hospital inpatients for final end‐user satisfaction. Full details are provided in Supporting Information: File S3. In summary, changes included the addition of a neutral response option and a question where patients reported when the most recent bedside handover occurred that they were reflecting on while completing the survey and major revisions to the response options for the construct ‘Level of patient participation in bedside handover’. The latter resulted in the decision for further inpatient testing. In Round 2, five inpatients completed the survey. Responses were positive, and no further changes were made to the survey.

### 2.4 Phase 3

#### 2.4.1 Study setting

This study was conducted on five wards in one Australian metropolitan tertiary hospital, including (1) gastrointestinal medicine and surgery; (2) cardiology medicine; (3) neurology medicine; (4) vascular medicine and surgery and (5) head and neck, urology and gynaecology surgery.

#### 2.4.2 Participants and procedure

Inclusion criteria were inpatients aged ≥18 years who had experienced ≥1 bedside handover. Exclusion criteria were patients physiologically unstable or not mentally capable of participation (as per healthcare professional judgement), or patients unable to read English. Research Assistants screened all patients on the ward on a given day of data collection and met with the Nurse Unit Manager or their delegate to determine participants who met the inclusion and exclusion criteria. Once eligible participants were identified, the Research Assistant approached the patient providing a written (ethics‐approved information sheet) and verbal description of the project. The survey was provided to the patient in paper format with a pen. Participants had the option to complete the survey independently. The Research Assistant returned at an agreed upon time to collect the survey. Some participants required assistance completing the survey, such as patients who were unable to write due to their injuries, the Research Assistant completed responses for these participants as required.

The minimum number of cases required for a ‘good’ sample and EFA is 300.^26^ We attempted to over recruit by 10% to account for surveys that would be returned incomplete or with invalid responses.

To support validity analyses, a sample of *N* = 40 nurses (who were involved in the same bedside handover patient participants most recently experienced) completed the process section of the ‘Quality improvement and audit tool for nurse‐to‐nurse bedside clinical handover in ward settings’.^27^ Permission was gained from the lead author before using the tool.

#### 2.4.3 Data collection

**Contextual and demographic information**

Participants were asked to report their age, gender and number of nights spent in the hospital during the current admission. Additionally, participants were asked questions about the bedside handover they were reflecting on when completing this survey including: ‘when did your most recent bedside handover happen?’ ‘handover was close to my bedside, so I could participate’, ‘a family/friend/carer/significant other was present during the most recent bedside handover’, ‘I felt well enough to participate during the most recent bedside handover’, ‘this was my first bedside handover (for any hospital admission)’ and ‘did you share your room with another patient during your most recent bedside handover?’.

**Patient participation in bedside handover**

Items (*n* = 42) generated and refined in Phase 1 and 2 of this study were presented to patients on a paper‐based survey. The survey had three sections relating to the three constructs in our conceptual framework. Participants were asked to think about a recent bedside handover they had experienced when completing the entire survey. To match the style of the items, two sections had the response options: 1 = *strongly disagree*, 2 = *disagree*, 3 = *neutral*, 4 = *agree* and 5 = *strongly agree* and one section had the response options: 1 = *not at all*, 2 = *slightly*, 3 = *moderately*, 4 = *considerably* and 5 = *a great deal* (see Supporting Information: File S4).

#### 2.4.4 Data analysis

The FACTOR programme and Mplus (v8) packages were used for all analyses. To explore the internal structural validity of the 42 items, EFA was conducted. EFA was appropriate since the instrument is new and its dimensional structure and therefore, the optimal scoring approach is unknown. The analyses adopted three foci: (1) evaluation of overall model fit for the factor solutions; (2) evaluation of item performance and (3) evaluation of the performance of each extracted factor. Please see Supporting Information: File S5 for full data analysis details. Due to the theoretical nature of the potential underlying constructs, it is expected that these will be orthogonal and therefore, an oblique rotation approach was used (Robust Promin).^28^

An intentionally large item pool was generated to maximize content domain coverage. Therefore, strict item retention criteria were adopted to eliminate poorly performing items and ultimately, minimize respondent burden when completing the final instrument. After extracting the most accommodating factor solution (seven‐factor) solution, item adequacy was appraised using multiple indicators. Items with low overall communality (*h* ^*2*^ < 0.30) with weak primary loadings (<0.63 using the benchmark of 0.63 as ‘very good’)^29^ or with close cross‐loading (<0.15 loading difference) on a secondary factor were eliminated.^30^ Because the factored item set influences the performance of each individual item, an iterative approach was taken to item deletion whereby the single worst performing item was removed at a time and the solution re‐estimated until a simple structure was achieved. The ultimate criterion guiding factor and item decisions will be substantive interpretability of the factor solution with consideration of content validity.

The resulting factors were assessed for their quality using the ‘Overall Reliability of fully‐Informative prior Oblique N‐EAP scores’ index (hereon ORION), which is an assessment of the reliability of the factor scores. ORION values >0.80 indicate the precision of the factor score estimates.^31^ The Factor Determinacy Index (hereon FDI) was also calculated to evaluate the accuracy of the factor score estimates given that these estimates in EFA are not unique.^32^ For completeness, and given its popularity, Cronbach's *α* was also computed and values >.80 taken to indicate ‘good’ internal consistency.

Finally, evidence for the construct validity of the factors was assessed with convergent correlation coefficients between patient and nurse reports, and converging patient demographics, such as age, gender, closeness of handover to bedside and patient rating of feeling ‘well’ enough to participate.

### 2.5 Consumer and clinician engagement

Recently, a systematic review of patient engagement in survey development studies identified that no surveys were coconstructed with patients from item development through to the validation phase.^33^ Thus, a purposeful collaboration between consumers, clinicians and researchers formed the basis of this study to ensure the survey was acceptable for end‐users and able to be translated into practice. The GRIPP2 short form was used to plan and report this study.^34^ Consumers, clinicians and researchers played an important and sustained role throughout the project from the commencement of ideas to item development and testing, through to analysis and the final write‐up of the findings (see Supporting Information: File S6 for full description). Consumer and clinical roles were planned using the ‘Patient Engagement Quality Guidance Tool’.^35^

‘The Partnership Analysis Tool’ was delivered to the consumer (M. S.), nurse (K. J.) and lead researcher (G. T.) every 6 months to monitor their partnership.^36^ Findings of the tool helped to develop a clear understanding of the purpose of the collaboration and reflect on ways to strengthen new and existing partnerships by engaging in discussions about issues and ways forward. Further details about the ‘The Partnership Analysis Tool’ and survey results are provided in Supporting Information: File S6. Overall, the partners consistently reached benchmarks that indicated they had a genuine collaboration.

## 3 RESULTS

From November 2020 to January 2021, 354 patients were approached for participation and provided with a survey, of those 326 returned completed surveys, representing a 92.1% response rate. Due to ethical requirements at the hospital, we were unable to ask patients why they declined, but patients that voluntarily provided this information indicated reasons, such as feeling too unwell, had visitors present, were not interested in participation or were frustrated with being in hospital. Participants had a mean age of 61.3 years (SD = 17.2 years), were in the hospital for a median of three nights (interquartile range = 4) and were mostly males (*n* = 185, 57.8%). The handover that patients reflected on when completing the survey was close to the bedside to enable participation (*n* = 260, 81.3%) and in private rooms (*n* = 244, 71.1%). For 266 (85.3%) patients, it was not their first handover and they felt well enough to participate (*n* = 299, 95.5%). A family/friend/carer/significant other was only present for 19 (6.1%) handovers.

Of the surveys returned, five were removed as they had ≥50% missing data. Of the 321 surveys retained in the final analyses, the missing values were 2.26%. Little's missing completely at random (MCAR) test was significant, indicating missing data was systematic *χ* ^2^ (2602, *n* = 321) = 2884.19, *p* < .001. Upon investigation, it appeared that participants were more likely to complete questions earlier in the survey than later, providing a possible reason for a significant MCAR test. To address missing data, a hot‐deck multiple imputation approach designed for use in EFA was used.^37^

### 3.1 Exploratory factor analysis

Horn's parallel analysis and Schwarz's (BIC) dimensionality text suggested a three‐factor solution, while the Hull method supported a unidimensional solution. Using all 42 items, Kaiser's criterion, which is known to over‐estimate the number of factors,^38^ identified the upper bound for the number of factors at seven. Additionally, only the seven‐factor solution achieved adequate model fit across all criteria (see Table 1). Based on these results, a range of factor solutions were explored from a unidimensional to a seven‐factor solution.

**Table 1:** Model fit indices will full item set (*k* = 42, *N* = 321)

*m* =	*χ* ^2^	Δ*χ* ^2^	*χ* ^2^/*df*	RMSEA (95% CI)	CFI	TLI	SRMR
2	3598.94*	NA	4.63	0.106 (0.103, 0.110)	0.928	0.920	0.087
3	2521.40*	**659.87** *	3.42	0.087 (0.083, 0.090)	**0.954**	0.947	**0.059**
4	2048.27*	**437.53** *	2.93	0.078 (0.074, 0.081)	**0.965**	**0.958**	**0.052**
5	1641.21*	**366.04** *	2.48	0.068 (0.064, 0.072)	**0.975**	**0.967**	**0.042**
6	1441.51*	**225.18** *	2.31	0.064 (0.060, 0.068)	**0.979**	**0.971**	**0.037**
7	1171.25*	**255.30** *	**1.99**	**0.056 (0.051, 0.060)**	**0.985**	**0.978**	**0.031**

*Note*: *χ* ^2^ tests the improvement of fit between the given solution and the one more restrictive solution (the row above it). For efficiency in computation and comparison, Mplus was used to compute these estimates.

Abbreviations: CFI, comparative fit index; CI, confidence interval; RMSEA, root mean square error of approximation; SRMR, standardized root mean squared error; TLI, Tucker–Lewis index.

* *p* < .001.

Decisions about a number of factors to extract were coincident with the analysis of item performance. Beginning with the most accommodating factor solution (seven factors), an iterative approach was taken to evaluate item performance using the criteria specified above. Items were removed one at a time, before re‐estimating the EFA to further interrogate items.

In the final solution, 26 items loading strongly and significantly (*p* < .05) onto three factors represented a statistically and theoretically sound solution. Keiser's criterion, Horn's parallel analysis and Schwarz's (BIC) dimensionality text converged in supporting the three‐factor solution. The Hull method, once again, favoured a unidimensional solution.

Following this item‐removal process, model fit for the three‐factor solution was excellent, _adjusted_ *χ* ^2^ (200) = 91.58, *p* = 999, *χ* ^2^/*df* = 0.44, root mean square error of approximation < 0.01 (95% confidence interval [CI]: [<0.01, 0.01]), comparative fit index > 0.99, Tucker–Lewis index = 0.1.01, standardized root mean squared error = 0.04. The three factors combined explained 80.6% of variance in the 24 items. Table 2 reports the robust Promin item loadings and factor quality information.

**Table 2:** Promin item loadings and factor quality information

S. no.	Items	Factor			*h* ^2^
		1	2	3	
1	I felt respected by nurses during bedside handover	0.996			0.64
2	I was confident talking with nurses during bedside handover	0.974			0.54
3	My privacy was maintained when sharing personal information during bedside handover	0.953			0.75
4	Nurses paid attention to me during bedside handover	0.945			0.81
5	The nurses were welcoming during bedside handover	0.916			0.91
6	The bedside handover discussion was loud enough for me to hear	0.891			0.85
7	Nurses responded to me during bedside handover	0.863			0.82
8	I felt informed enough to participate in bedside handover	0.842			0.66
9	Nurses listened to me during bedside handover	0.826			0.88
10	There was enough time for nurse‐patient discussions during bedside handover	0.733			0.89
11	I could understand the words nurses used during bedside handover	0.675			0.72
12	How much did you share information relevant to you during bedside handover?		0.916		0.74
13	How much did you share information relevant to your healthcare during bedside handover?		0.892		0.86
14	How much did you ask questions during bedside handover?		0.870		0.84
15	How much did you participate in planning during bedside handover?		0.665		0.50
16	I felt more in control of my healthcare, after bedside handover			0.973	0.82
17	I felt more involved in my healthcare, after bedside handover			0.958	0.84
18	I felt more confident in my healthcare, after bedside handover			0.950	0.89
19	Bedside handover made me feel more valued as a person			0.926	0.90
20	My relationship with nurses has improved, after bedside handover			0.911	0.84
21	I trusted my nurses more, after bedside handover			0.894	0.75
22	Bedside handover helped me understand what was happening with my care			0.853	0.73
23	I felt more informed about my healthcare, after bedside handover			0.843	0.73
24	Overall, I felt satisfied with bedside handover		0.331	0.652	0.80
	*λ*	15.21	2.12	1.36	*M* = 0.78
	Variance	8.57	2.96	7.16	SD = 0.11
	ORION	0.98	0.94	0.98	
	FDI	0.99	0.97	0.99	
	H‐observed	0.88	0.88	0.92	

*Note*:  loadings < weak loadings < 0.30 have been suppressed.

Abbreviation: FDI, Factor Determinacy Index.

The three factors closely aligned with our initial conceptual framework of patient participation in bedside handover, and thus labels used in our conceptual framework were used to name factors. Based on an interpretation of the content of the highest loading items, Factor 1 represents conditions for patient participation in bedside handover. Factor 2 represents the level of patient participation in handover, particularly with respect to the communication. Finally, Factor 3 represents the evaluation of patient participation in bedside handover.

### 3.2 Factor reliability

Table 2 shows the three retained factors are supported by strong evidence for their reliability with all ORION estimates > 0.80, all FDI > 0.90, and all replicability estimates (H‐observed) >0.80). For comparison, the Cronbach's *α* estimates are good to excellent as follows: Factor 1 = 0.96 (95% CI: [0.95, 0.96]), Factor 2 = 87 (95% CI: [0.85, 0.90]) and Factor 3 = 96 (95% CI: [0.95, 0.96]).

### 3.3 Convergent and divergent construct validity

The interfactor correlations were significant and strong, corroborating the use of an oblique rotation approach. Factors 1 and 2 were strongly correlated, *r* = .53, 95% CI: [0.43, 0.64]. Factors 2 and 3 were very strongly correlated, *r* = .78, 95% CI: [0.70, 0.84]. Finally, Factors 1 and 3 were strongly correlated, *r* = .63, 95% CI: [0.26, 0.54]. As these three dimensions are theoretically linked, these strong associations provide evidence for the convergent validity of the scales. Discriminant validity for each factor is evidenced by the larger average item‐factor loading (F1 = 0.87, F2 = 0.84 and F3 = 0.88) relative to the average interfactor correlations (F1= 0.65, F2 = 0.58 and F3 = 0.70).

Patient reports on the three extracted factors were regressed onto a group of nurse respondent questions to determine if patient reports could be externally corroborated. These results show very high and significant overlap between patient report of ‘conditions for patient participation in bedside handover’ (*R* ^2^ = .94, *p* < .001), ‘level of patient participation in bedside handover’ (*R* ^2^ = .82, *p* < .001) and ‘evaluation of patient participation in bedside handover’ (*R* ^2^ = .75, *p* < .001) and nurse observer reports.

Participants provided much higher ratings of ‘conditions for patient participation in bedside handover’, ‘level of patient participation in bedside’ and ‘evaluation of patient participation in bedside handover’ when handover occurred close to their bedside compared with participants who reported handover was not close to their bedside. The statistics for these group comparisons are reported in Table 3.

**Table 3:** Descriptive and inferential statistics for mean comparisons across the location of bedside handover

	Close to bedside	Far from bedside	
	*M*, SD	*M*, SD	a *F*(*df*), *p*, *d*
Conditions for patient participation	4.30, 0.60	3.32, 0.91	(1, 58.12) = −7.28, *p* < .001, *d* = −1.26)
Level of patient participation	3.00, 1.06	2.01, 1.19	(1, 66.75) = −5.40, *p* < .001, *d* = −0.86
Evaluation of patient participation	4.09, 0.65	3.13, 1.12	(1, 53.23) = −5.76, *p* < .001, *d* = −1.05

^a^ All *F* estimates utilize the Welch correction for violation of equality of variance assumption.

Participants provided much higher ratings of ‘conditions for patient participation in bedside handover’ (*M* = 4.16, SD = 0.74, *F*[1, 301] = −3.62, *p* < .001, *d* = −1.07) and moderately higher ratings of ‘level of patient participation in bedside handover’ (*M* = 2.85, SD = 1.14, *F*[1, 289] = −2.50, *p* = .013, *d* = −0.69) when they reported they were well enough to participate compared with participants who reported they were not well enough to participate (‘conditions for patient participation in bedside handover’ *M* = 3.37, SD = 0.83; ‘level of patient participation in bedside handover’ *M* = 2.07, SD = 1.07). As might be expected, the evaluations of benefits from bedside handover did not vary as a function of patients self‐rated health status (healthy enough *M* = 3.95, SD = 0.81 vs. not healthy enough *M* = 3.55, SD = 0.88), *F*[1, 297] = −1.76, *p* = .080, *d* = −0.50.

As displayed in Table 4, ratings on the factors did not vary as a function of gender or whether the patient shared a room. Likewise, none of the three factors varied as a function of patient age (all *r*s < .05 all *p*s > .437). The number of nights the patient had stayed in the hospital correlated weakly with ‘conditions for patient participation in bedside handover’ (*r* = −.16, *p* = .004), however, did not correlate with ‘level of patient participation in bedside handover’ or ‘evaluation of patient participation in bedside handover’ (all *r*s < −.08, all *p*s > .180).

**Table 4:** Descriptive and inferential statistics for mean comparisons across gender and sharing a room status

	Conditions for patient participation in bedside handover				Level of patient participation in bedside handover				Evaluation of patient participation in bedside handover			
	*M*	SD	*F*(*df*), *p*	*d*	*M*	SD	*F*(*df*), *p*	*d*	*M*	SD	*F*(*df*), *p*	*d*
Male	4.14	0.70	0.17(1,249.02^a^), .865	0.02	2.84	1.10	0.74(1,303), .461	0.09	3.96	0.71	0.73(1,218.53^a^), .461	0.09
Female	4.13	0.83			2.75	1.20			3.89	0.94		
Did not share room	4.11	0.80	−0.66(1,303), .507	−0.08	2.80	1.12	−0.31(1,300), .759	−0.04	3.93	0.85	0.09(1,299), .926	0.01
Shared room	4.18	0.63			2.84	1.21			3.92	0.74		
	*r*	*p*	–	–	*r*	*p*	–	–	*r*	*p*	–	–
Age	.014	.810			.045	.437			.032	.577		

^a^ Welch adjustment is applied when the test of equal variance was violated (Levene's test < 0.05).

## 4 DISCUSSION

We developed the patient participation in bedside handover survey, a 24‐item survey, comprising three reliable factors, which supports our initial conceptual framework of patient participation in bedside handover (see Supporting Information: File S7). We found strong correlations between factors, which extends our theoretical understanding of patient participation in bedside handover by confirming that nurse activation of the right conditions for patient participation strongly predicts patient behaviour, and their perceived outcomes of handover. These factors were cross‐validated by significant and strong overlap with observer (nurse) reports and external conditions such as whether handover occurred close to the patient's bedside.

In our study, many nurse disposition items were retained in ‘conditions for patient participation in bedside handover’, and whether patients' perceived nurses were close to the bedside strongly influenced this factor. This confirms the importance of nurses' facilitating behaviours as critical to the success of patient participation in handover. Other researchers have shown that nurses can control patient participation by displaying or not displaying inviting actions,^39^ including whether they choose to conduct handover close to the bed or not.^9^, ^10^

Notably, fewer items about patient conditions loaded on all factors. Researchers have demonstrated that patients have a strong preference for handover to occur at the bedside,^40^ and view being involved in bedside handover as their right.^41^ Thus, we suspect there was a low variation on items, such as ‘I want handover to take place at my bedside, with me present’ and ‘I think bedside handover is important’, resulting in their removal. Additionally, patients' desire for information via bedside handover often far outweighs their concerns about confidentiality,^7^, ^41^ with recent research showing that no patients in semiprivate rooms refused bedside handover.^42^, ^43^ This was supported by our convergent/divergent construct validity analyses showing that being in a shared room had no effect on the factors. Importantly, none of the factors varied as a function of participant age and gender, suggesting all three factors are independent of these important demographic characteristics. We propose that there may be little additional value in measuring patient conditions and demographics, given patients tend to be overwhelmingly in support of bedside handover occurring. However, it should be noted that whether patients felt well enough to participate covary with ‘conditions for patient participation in bedside handover’ and ‘level of patient participation in bedside handover’, suggesting this is an important patient condition to assess to tailor bedside handover to the patient's needs.

Retained items for ‘level of patient participation in bedside handover’ related to patient information‐exchanging behaviours. This is consistent with evidence from other Australian and Swedish patients who rank being able to speak as one of the most important features of bedside handover.^18^, ^40^ But ‘level of patient participation in bedside handover’ may be a less complex construct than initially conceptualized. Items related to patient behaviours varied more and therefore exhibited better loadings than items related to patient preferences, which did not meet stringent item retention criteria. Given preferences for bedside handover do not vary much, we suggest they might not be theoretically or empirically useful to measure. Other researchers have demonstrated that patient preferences for and reported behaviours of patient participation are often in concordance,^44^, ^45^ further supporting our survey that only measures behaviours.

All items that we generated for the construct ‘evaluation of the benefits of patient participation in bedside handover’ were retained. This confirms that the perceived outcomes that were developed sufficiently covary and represent the variation in patient perceptions of outcomes of handover. As systematic review findings suggest, both individual benefits for patients, like feeling involved and confident, and relational benefits, like having improved relationships and trust with nurses, were items retained in our survey.^46^ Currently, the efficiency of one type of handover over another (i.e., bedside handover, tape‐recorded handover, verbal handover and written handover) is yet to be established.^47^ However, quantitative research measuring bedside handover effectiveness is starting to emerge, showing that while bedside handover influenced nurses' recognition of patient participation, patients did not experience any effects; however, surveys used measured patient empowerment, quality of care and individualized care.^48^ Thus, ‘evaluation of patient participation in bedside handover’ is an important factor in our survey because it directly measures outcomes related to patient participation in bedside handover and could assist in building evidence of outcomes.

### 4.1 Reflection on consumer and clinician engagement

As per the GRIPP2 checklist, the consumer, nurse and lead researcher reflected on the process of consumer and clinician engagement.^34^ We believe that consumer and clinician engagement in survey development influenced the great feedback about the content, design and importance of the survey during end‐user satisfaction testing; findings reported by other researchers.^49^ Additionally, the strong partnerships built during this study have led to further collaborations between the consumer, nurse and lead researcher. We used a survey to monitor our partnership over the course of the study, which we viewed as invaluable. Our consumer particularly liked how the survey established willingness and commitment to the research and then provided a way to make sure that the enthusiasm for partnering was not lost over the course of the project. The lead researcher liked that slight fluctuations in survey scores allowed her to respond. For example, during the COVID‐19 pandemic when research delays occurred and communication patterns changed, survey results heightened her awareness of issues and enabled the implementation of strategies to maintain partnerships. However, we acknowledged that the survey we selected is aimed at ‘health promotion’ partnerships. While the survey was satisfactory, in future research we would spend more time deciding which partnership survey is fit‐for‐purpose, utilizing a database like the Centre of Excellence on Partnership with Patients and the Public's^50^ ‘Evaluation Toolkit’.

### 4.2 Clinical implications

Our study highlights that nurses' behaviours are a core condition for active patient participation in handover and patients' perceived outcomes. Educators for undergraduate nurses and nurses in the clinical workforce should provide training for nurses to provide them with the skills to promote patient engagement. Items in ‘conditions for patient participation in bedside handover’ could guide training on important facilitating behaviours to entrench in nurses, while ‘level of patient participation in bedside handover’ could guide training on ways to engage patients in handover. In novel work by Barello et al.,^51^ they have developed nurse education training in patient engagement strategies training that gives nurses practical patient engagement skills, providing guidance for others.

There are many future directions for the survey. We know that routine communication encounters like handover are not always used to their full potential to promote meaningful patient participation.^52^ The survey could be used alongside hospital observational audits, to provide another source of rich patient‐focussed feedback and benchmark bedside handover performance over time. Further, change managers and researchers are increasingly improving and implementing bedside handover in practice.^7^ Our robust survey can be used as a tool to measure the effectiveness of these efforts, from the patient perspective. However, ongoing survey refinement is warranted as high correlations between items could indicate the need for further item reduction. Additionally, a 24‐item survey could be burdensome for hospitalized patients, thus reducing items is further supported. Unfortunately, we did not collect data on how many participants required assistance completing the survey. Future researchers should collect these data to identify how burdensome the survey is for hospitalized patients and to allow appropriate planning for survey dissemination.

## 5 CONCLUSIONS

Our team of consumers, nurses and researchers rigorously developed a survey to measure patient perceptions of participation in bedside handover. This survey has the potential to increase the quality of evaluation of bedside handover implementation and improvement efforts. Using a systematic and partnership design, we reduced the items from 130 to a more precise set of 24 that represent three valid and highly reliable constructs. Our understanding of the concept of patient participation in bedside handover has been clarified as a result of the factors that have emerged. For example, we found less value in measuring patient conditions for bedside handover, while the importance of nurses' facilitating behaviours for reaping handover benefits cannot be understated. Additionally, there is variation in how patients engage in handover, including sharing information, asking questions and planning care, an important consideration for tailoring care to the individual patient. Involving consumers and nurses in all phases of this research study strengthened the survey developed; we recommend that research teams monitor consumer and clinician engagement throughout the research study to ensure true engagement in the research process occurs.

## AUTHOR CONTRIBUTIONS

*The conception and design of the study*: Georgia Tobiano, Andrea P. Marshall, Kim Jenkinson, Margaret Shapiro, Michael Ireland. *Acquisition of data*: Therese Gardiner. *Analysis and interpretation of data*: Georgia Tobiano, Andrea P. Marshall, Therese Gardiner, Kim Jenkinson, Margaret Shapiro, Michael Ireland. *Drafting the article or revising it critically for important intellectual content*: Georgia Tobiano, Andrea P. Marshall, Therese Gardiner, Kim Jenkinson, Margaret Shapiro, Michael Ireland. *Final approval of the version to be submitted*: Georgia Tobiano, Andrea P. Marshall, Therese Gardiner, Kim Jenkinson, Margaret Shapiro, Michael Ireland.

## CONFLICT OF INTEREST

The authors declare no conflict of interest.

## Supporting information

Supplementary information.

## Data Availability

Participants of this study did not agree for their data to be shared publicly, so supporting data is not available. Upon reasonable request, our research team could approach the approving ethics committee to seek opportunities for data sharing.

## References

[1] Makary MA , Daniel M . Medical error—the third leading cause of death in the US. BMJ. 2016;353:e1‐e5. 10.1136/bmj.i2139%J.BMJ 10.1136/bmj.i2139 27143499

[2] The Joint Commission . *Sentinel event data: Root causes by the event type: 2004‐June 2013*. Accessed November 5, 2016. http://www.medleague.com/wp-content/uploads/2013/11/Root_Causes_by_Event_Type_2004-2Q2013.pdf

[3] Anderson J , Malone L , Shanahan K , Manning J . Nursing bedside clinical handover—an integrated review of issues and tools. J Clin Nurs. 2015;24(5‐6):662‐671. 10.1111/jocn.12706 25319724

[4] Mardis T , Mardis M , Davis J , et al. Bedside shift‐to‐shift handoffs: a systematic review of the literature. J Nurs Care Qual. 2016;31(1):54‐60. 10.1097/NCQ.0000000000000142 26192148

[5] Sherman J , Sand‐Jecklin K , Johnson J . Investigating bedside nursing report: a synthesis of the literature. Medsurg Nurs. 2013;22(5):308‐318. 24358572

[6] Longtin Y , Sax H , Leape LL , Sheridan SE , Donaldson L , Pittet D . Patient participation: current knowledge and applicability to patient safety. Mayo Clin Proc. 2010;85(1):53‐62. 10.4065/mcp.2009.0248 20042562 PMC2800278

[7] Tobiano G , Bucknall T , Sladdin I , Whitty JA , Chaboyer W . Patient participation in nursing bedside handover: a systematic mixed‐methods review. Int J Nurs Stud. 2018;77:243‐258. 10.1016/j.ijnurstu.2017.10.014 29149634

[8] Tobiano G , Marshall A , Bucknall T , Chaboyer W . Patient participation in nursing care on medical wards: an integrative review. Int J Nurs Stud. 2015;52(6):1107‐1120. 10.1016/j.ijnurstu.2015.02.010 25769475

[9] Street M , Dempster J , Berry D , et al. Enhancing active patient participation in nursing handover: a mixed methods study. J Clin Nurs. 2021;31(7‐8):1016‐1029. 10.1111/jocn.15961 34268829

[10] Malfait S , Eeckloo K , Van Biesen W , Deryckere M , Lust E , Van Hecke A . Compliance with a structured bedside handover protocol: an observational, multicentred study. Int J Nurs Stud. 2018;84:12‐18. 10.1016/j.ijnurstu.2018.04.011 29729557

[11] Thórarinsdóttir K , Kristjánsson K . Patients' perspectives on person‐centred participation in healthcare: a framework analysis. Nurs Ethics. 2014;21(2):129‐147. 10.1191/0969733006nej898oa 23812560

[12] Tobiano G , Marshall A , Bucknall T , Chaboyer W . Activities patients and nurses undertake to promote patient participation. J Nurs Scholarsh. 2016;48(4):362‐370. 10.1111/jnu.12219 27198079

[13] Fleischer S , Berg A , Zimmermann M , Wüste K , Behrens J . Nurse‐patient interaction and communication: a systematic literature review. J Public Health. 2009;17(5):339‐353. 10.1007/s10389-008-0238-1

[14] Evans EC . Exploring the nuances of nurse‐patient interaction through concept analysis: impact on patient satisfaction. Nurs Sci Q. 2016;29(1):62‐70. 10.1177/0894318415614904 26660778

[15] Köberich S , Reimann C . Development and psychometric testing of a questionnaire on perceived participation in the nursing handover at the bedside of hospital patients on a normal ward: a cross sectional study. Klinische Pflegeforschung. 2017;3:72‐84.

[16] Timonen L , Sihvonen M . Patient participation in bedside reporting on surgical wards. J Clin Nurs. 2000;9(4):542‐548. 10.1046/j.1365-2702.2000.00400.x 11261134

[17] Tobiano G , Bucknall T , Marshall A , Guinane J , Chaboyer W . Patients' perceptions of participation in nursing care on medical wards. Scand J Caring Sci. 2015;30(2):542‐548. 10.1111/scs.12237 26036723

[18] Whitty JA , Spinks J , Bucknall T , Tobiano G , Chaboyer W . Patient and nurse preferences for implementation of bedside handover: do they agree? Findings from a discrete choice experiment. Health Expect. 2016;20(4):742‐750. 10.1111/hex.12513 27804191 PMC5512991

[19] Friesen MA , Herbst A , Turner JW , Speroni KG , Robinson J . Developing a patient‐centered ISHAPED handoff with patient/family and parent advisory councils. J Nurs Care Qual. 2013;28(3):208‐216. 10.1097/NCQ.0b013e3182868c9c 23528749

[20] Herbst AM , Friesen MA , Karen GS . Caring, connecting, and communicating: reflections on developing a patient‐centered bedside handoff. Int J Hum Caring. 2013;17(2):23‐28. 10.20467/1091-5710.17.2.16

[21] Kassean HK , Jagoo ZB . Managing change in the nursing handover from traditional to bedside handover—a case study from Mauritius. BMC Nurs. 2005;4(1):e1‐e6. 10.1186/1472-6955-4-1 PMC548693 15676078

[22] Maxson PM , Derby KM , Wrobleski DM , Foss DM . Bedside nurse‐to‐nurse handoff promotes patient safety. Medsurg Nurs. 2012;21(3):140‐145. 22866433

[23] Sand‐Jecklin K , Sherman J . A quantitative assessment of patient and nurse outcomes of bedside nursing report implementation. J Clin Nurs. 2014;23(19/20):2854‐2863. 10.1111/jocn.12575 24606553

[24] Wakefield DS , Ragan R , Brandt J , Tregnago M . Making the transition to nursing bedside shift reports. Jt Comm J Qual Patient Saf. 2012;38(6):243‐253. 10.1016/s1553-7250(12)38031-8 22737775

[25] Polit DF , Beck CT . The content validity index: are you sure you know what's being reported? Critique and recommendations. Res Nurs Health. 2006;29(5):489‐497. 10.1002/nur.20147 16977646

[26] Pett MA , Lackey NR , Sullivan JJ . Making Sense of Factor Analysis: The Use of Factor Analysis for Instrument Development in Health Care Research. Sage Publications; 2003.

[27] Redley B , Waugh R . Mixed methods evaluation of a quality improvement and audit tool for nurse‐to‐nurse bedside clinical handover in ward settings. Appl Nurs Res. 2018;40:80‐89. 10.1016/j.apnr.2017.12.013 29579504

[28] Lorenzo‐Seva U , Ferrando PJ . Robust promin: a method for diagonally weighted factor rotation. Rev de Psicol. 2019;25(1):99‐106. 10.24265/liberabit.2019.v25n1.08

[29] Comrey AL , Lee HB . A First Course in Factor Analysis. 2nd ed. Academic Press; 1992.

[30] Tabachnick BG , Fidell LS . Using Multivariate Statistics. 5th ed. Allyn & Bacon; 2007.

[31] Ferrando PJ , Lorenzo‐Seva U . Assessing the quality and appropriateness of factor solutions and factor score estimates in exploratory item factor analysis. Educ Psychol Meas. 2018;78(5):762‐780. 10.1177/0013164417719308 32655169 PMC7328234

[32] Ferrando PJ , Lorenzo‐Seva U . Assessing score determinacy, measurement quality, and closeness to unidimensionality in exploratory item factor analysis. Educ Psychol Meas. 2017;78(5):762‐780. 10.1177/0013164417719308 32655169 PMC7328234

[33] Clavel N , Paquette J , Dumez V , et al. Patient engagement in care: a scoping review of recently validated tools assessing patients' and healthcare professionals' preferences and experience. Health Expect. 2021;24(6):1924‐1935. 10.1111/hex.13344 34399008 PMC8628592

[34] Staniszewska S , Brett J , Simera I , et al. GRIPP2 reporting checklists: tools to improve reporting of patient and public involvement in research. BMJ. 2017;358:e1‐e7. 10.1136/bmj.j3453 PMC5539518 28768629

[35] Patient Focused Medicine Organisation . Patient engagement quality guidance tool. Accessed October 13, 2018. http://patientfocusedmedicine.org/peqg/patient-engagement-quality-guidance-scenario-1.pdf

[36] VicHealth . The partnerships analysis tool. Accessed December 21, 2021. https://www.vichealth.vic.gov.au/media-and-resources/publications/the-partnerships-analysis-tool

[37] Lorenzo‐Seva U , Van Ginkel JR . Multiple imputation of missing values in exploratory factor analysis of multidimensional scales: estimating latent trait scores. An Psicol. 2016;32(2):596‐608. 10.6018/analesps.32.2.215161

[38] Nunnally JC . Psychometric Theory. 3rd ed. McGraw‐Hill; 1994.

[39] Forde MF , Coffey A , Hegarty J . Bedside handover at the change of nursing shift: a mixed‐methods study. J Clin Nurs. 2020;29(19‐20):3731‐3742. 10.1111/jocn.15403 32644255

[40] Oxelmark L , Whitty JA , Ulin K , Chaboyer W , Oliveira Gonçalves AS , Ringdal M . Patients prefer clinical handover at the bedside; nurses do not: evidence from a discrete choice experiment. Int J Nurs Stud. 2020;105:103444. 10.1016/j.ijnurstu.2019.103444 32200099

[41] Bressan V , Cadorin L , Stevanin S , Palese A . Patients experiences of bedside handover: findings from a meta‐synthesis. Scand J Caring Sci. 2019;33(3):556‐568. 10.1111/scs.12673 30866081

[42] Malfait S , Van Hecke A , Van Biesen W , Eeckloo K . Is privacy a problem during bedside handovers? A practice‐oriented discussion paper. Nurs Ethics. 2019;26(7‐8):2288‐2297. 10.1177/0969733018791348 30134750 PMC7323750

[43] McCloskey RM , Furlong KE , Hansen L . Patient, family and nurse experiences with patient presence during handovers in acute care hospital settings: a systematic review of qualitative evidence. JBI Evid Synth. 2019;17(5):754‐792. 10.11124/JBISRIR-2017-003737 30889068

[44] Martinsson C , Uhlin F , Wenemark M , Eldh AC . Preference‐based patient participation for most, if not all: a cross‐sectional study of patient participation amongst persons with end‐stage kidney disease. Health Expect. 2021;24(5):1833‐1841. 10.1111/hex.13323 34337836 PMC8483194

[45] Tobiano G , Chaboyer W , Dornan G , Teasdale T , Manias E . Older patients' engagement in hospital medication safety behaviours. Aging Clin Exp Res. 2021;33(12):3353‐3361. 10.1007/s40520-021-01866-3 33945114

[46] Clari M , Conti A , Chiarini D , Martin B , Dimonte V , Campagna S . Barriers to and facilitators of bedside nursing handover: a systematic review and meta‐synthesis. J Nurs Care Qual. 2021;36(4):e51‐e58. 10.1097/ncq.0000000000000564 33852530

[47] Bakon S , Wirihana L , Christensen M , Craft J . Nursing handovers: an integrative review of the different models and processes available. Int J Nurs Pract. 2017;23(2):e1‐e8. 10.1111/ijn.12520 28176414

[48] Malfait S , Eeckloo K , Van Biesen W , Van Hecke A . The effectiveness of bedside handovers: a multilevel, longitudinal study of effects on nurses and patients. J Adv Nurs. 2019;75(8):1690‐1701. 10.1111/jan.13954 30666713

[49] Brett J , Staniszewska S , Mockford C , et al. Mapping the impact of patient and public involvement on health and social care research: a systematic review. Health Expect. 2014;17(5):637‐650. 10.1111/j.1369-7625.2012.00795.x 22809132 PMC5060910

[50] Centre of Excellence on Partnership with Patients and the Public . Evaluation toolkit. Accessed December 20, 2021. https://ceppp.ca/en/evaluation-toolkit/

[51] Barello S , Graffigna G , Pitacco G , Mislej M , Cortale M , Provenzi L . An educational intervention to train professional nurses in promoting patient engagement: a pilot feasibility study. Front Psychol. 2016;7:2020. 10.3389/fpsyg.2016.02020 28119644 PMC5222845

[52] Tobiano G , Jerofke‐Owen T , Marshall AP . Promoting patient engagement: a scoping review of actions that align with the interactive care model. Scand J Caring Sci. 2021;35(3):722‐741. 10.1111/scs.12914 33068042

