# Supplementary material for: Development and psychometric testing of the patient participation in bedside handover survey
Source: Health Expect. 2022 Jul 27;25(5):2492–502. doi: 10.1111/hex.13569 (PMC9615084; doi:10.1111/hex.13569)
Supplement: Supplementary file 1 — Supplementary information. [file HEX-25--s001.docx]

**SUPPLEMENTARY FILES**

**Supplementary File 1:** Conceptual Framework Development

The conceptual framework was generated based on theory and empirical literature. First theory was identified; conceptual models of nurse-patient communication by Fleischer et al.^1^ and Evans^2^ were selected. Our team unanimously agreed that these conceptual models were a good fit to guide our conceptual framework development because bedside handover creates the conditions for ‘nurse-patient communication’ to occur. Four themes central to nurse-patient communication were identified:

1. The interpersonal style of the nurse is an antecedent for nurse-patient communication. Interpersonal communication can be verbal or non-verbal and is characterised by nurses’ friendliness, explanation, and mutuality, as well as nurses being supportive, empathetic, respectful, and present, and listening to the patient.
2. Successful nurse-patient communication must be adapted to the individual. There needs to be negotiation of patient preferences and their expectations must be met. Patients’ individual characteristics and personal life situation must also be considered.
3. The act of nurse-patient communication occurs when there is at least two people (nurse and patient) and these parties exchange information about the patient’s health. The intention of communication is ultimately to influence patient health and well-being and build patient understanding. The content is dynamic and both parties bring their own knowledge and goals to the encounter. The level of patient involvement in nurse-patient communication can range from passive to active.
4. Good nurse-patient communication results in positive outcomes like perceived quality of the interaction, the nurse-patient relationship, adaptation to the individual and patient understanding, many of which are a precursor for patient satisfaction.

We returned to our systematic review on patient participation in bedside handover^3^. This helped us to understand nurse-patient communication theory, in the context of bedside handover, and guided names and descriptions for our conceptual framework. We combined the theoretical and empirical (systematic review) data, to develop a conceptual framework for patient participation in bedside handover. This framework contains three constructs, which are defined below (See Figure 1):

**Figure 1.** Conceptual framework for patient participation in bedside handover

1. **Conditions for patient participation in bedside handover:** A state where both patients and nurses have met the conditions for active patient participation in bedside handover. Nurse conditions include their interpersonal style and behaviours that encourage patient participation and their information-sharing behaviours that need to be adapted to the individual. Patient conditions include their individual characteristics such as their capacity to participate in bedside handover.
2. **Level of patient participation in bedside handover:** an evaluation of levels of patient behaviour and preferences. For behaviour, it is the extent of participation a patient undertakes in bedside handover. Patient participation includes nurse-patient communication about health information, and the levels range from passive to active patient participation. For preference, it is an assessment of their preferred levels of participation. Health information includes topics like patient symptoms, capabilities or usual regimens, and patient may communicate by sharing information and asking questions. Patients may also participate non-verbally, for example by listening to nurse communication.
3. **Evaluation of patient participation in bedside handover:** An assessment of whether the level of participation a patient experienced was what they desired and resulted in positive outcomes like perceived quality of the interaction, the nurse-patient relationship, adaptation to the individual, patient understanding, and patient satisfaction.

We acknowledge that patient-centred care (PCC) has important overlap with nurse-patient communication, however, PCC is a broader concept encompassing more dimensions than nurse-patient communication. In addition, nurse-patient communication is likely a dimension of PCC. Thus, our conceptual framework is underpinned by PCC philosophy.

**Supplementary File 2:** Content validity

Content validity was assessed by an expert panel (n=16), including consumer advisors (n=4), nurses (n=4), and national and international researchers (n=4). We perceived that consumers, nurses and researchers have content expertise because they have experienced, practiced or published papers on bedside handover. Content experts were purposefully selected based on the knowledge they had on the topic of bedside handover. Our research team used our networks to identify consumers, clinicians and researchers with expertise in bedside handover. In Round 1 and 2 experts were invited to participate via email. They received the survey, an ethics approved participant information sheet and instructions for completing the survey. One reminder email was sent. Content experts were provided the option to complete content validity assessments face-to-face with a researcher or return via email.

In Round 1, all 16 experts that were invited to participate agreed to participate. In Round 2, three experts from Round 1 were invited and participated; one consumer, one nurse and one researcher. Experts were asked to evaluate the relevance of 60 individual items to the underlying construct on a 4-point scale (1 = “not relevant”, 2 = “somewhat relevant”, 3 = “quite relevant”, 4 = “very relevant”)^4^. Experts were also asked “was this item easy to understand” (1= yes, 0=no). Experts were encouraged to comment on ways to make the items more relevant and/or understandable. Additionally, they were asked to list additional items that were missing, to ensure the construct was fully covered.

The content validity index (CVI) was used to measure content validity^4^. CVI can be measured at the item level (I-CVI) and at the scale level (S-CVI)^4^. To compute I-CVI, the number of experts rating relevance as 3 or 4 was divided by the total number of experts. When there are six of more experts, the desired I-CVI at the item level is 0.78 or higher, indicating an item was relevant and retained^4^. Items scoring below 0.78 were considered for deletion or revision in conjunction with expert scores for understandability and comments.

The S-CVI provides a measurement of the average of the I-CVIs for all items for each construct^4^. S-CVI is calculated by summing the I-CVIs for each item and dividing it by the number of items^4^. A S-CVI of 0.80 or higher is deemed acceptable^4^.

Thirty-eight items had I-CVIs 0.78 or higher in Round 1. Of these 38 items, seven were retained unchanged, 16 were retained and reworded based on expert feedback and 15 were removed because expert comments revealed that they had high overlap with other items (that had high I-CVIs), and the other items had higher understandability ratings. Of the 22 items with low I-CVIs, 13 were removed immediately, while 9 were reworded based on expert feedback.

Ten questions were added based on expert feedback. In construct 2 items were about patient level of participation enacted. Experts suggested to add preference questions that matched these behaviour items. The behaviour question about sharing information was split into two items for more depth including “share information that is relevant to me” and “share information that is relevant to my healthcare”. Overwhelming, people suggested questions be added about family/friends/carers involvement in bedside handover, so the question was added: “before starting, nurses asked me if I wanted family/friends/carers/ significant others to participate in bedside handover” was added to construct 1. Many nurses wanted an armband question added, suggesting this is an important part of the introductions in a bedside handover so “nurses checked my armband during bedside handover” was added.

In Round 2, 42 items were presented to the experts. The consumer did not complete to survey, but informed our research team via email that she was happy with the content and suggested no changes. Three items had I-CVIs lower than 0.78, with only one expert finding all three items not relevant. These three items were discussed with our research team and were retained.

S-CVIs in Round 1 were 0.80 for construct 1, 0.81 for construct 2, and 0.78 for construct 3. After Round 2, S-CVIs improved to 1.0 for construct 1, 0.93 for construct 2 and 0.84 for construct 3.

**Supplementary File 3:** End-user satisfaction

Inclusion criteria were inpatients aged ≥ 18 years who had experienced ≥1 bedside handover. Exclusion criteria were patients physiologically unstable or not mentally capable of participation (as per healthcare professional judgment), or patients unable to read English. Purposive maximum variation sampling was used to identify patients that varied is terms of age and reason for admission (i.e. medical or surgical admission). The survey was delivered to participants with a researcher present. The researcher encouraged the participant to talk aloud when completing the survey and kept fieldnotes about their comments (TG). On survey completion the researcher asked the participants:

1. Were the survey instructions easy to understand?
2. Were the questions clear and easy to understand?
3. Were the answer options suitable?
4. Is there any thing missing?
5. Is there any way we can improve the way the survey looks, such as how it is laid out or size of the words?
6. Are there any changes you suggest?

In Round 1, 10 patients completed the survey, from the gastrointestinal medicine and surgery and cardiology medicine ward (same wards included in Phase 3 of this study). Six (60%) were females, with a median age of 68 years (IQR=30) and median length of stay of 4 nights in hospital (IQR 13.25). The instructions were viewed as easy to understand, with minor wording changing made to one sentence. Participants could not identify any missing items and liked the way the survey looked. The main changes were suggested in response to question 2 and 3, from the list above. Participants identified one double-barrelled item and one negatively worded item, that they preferred to be positive. Overwhelmingly patients wanted a neutral response option, thus the 4-item scale was changed to a 5-item scale. When delivering the survey, we noticed that patients reflected on past bedside handovers from differing timepoints, thus we added a question to identify which bedside handover they were thinking about when completing they survey. All of this feedback was reviewed, and decisions were made by the whole research team during a meeting.

Construct #2 in the survey included items about patients’ preferred behaviours and actual reported behaviours. Participants found that these questions were worded in a way that was difficult to understand and required a lot of researcher assistance to complete. Originally participants were presented with a stem for example “ask questions” and then presented with two items in relation to this stem for example “I wanted to do this” and “I needed to do this”. Overwhelmingly they wanted the stems to be more descriptive, and clarity around whether this behaviour related to themselves or the nurses. Additionally, participants found the response options inappropriate in this section, as they did not capture whether the behaviour was enacted. A different response scale was provided for this section (1=not at all, 2=slightly, 3= moderately, 4=considerably and 5=a great deal). Major revisions were made to this section, and it was determined that further inpatient testing was required.

In Round 2 five inpatients completed the survey. Three (60%) were female with a median age of 50 years (IQR=29) and had a median of 4 nights in hospital (IQR=11). Responses to all six questions were positive and no further changes were made to the survey.

**Supplementary File 4**: Survey delivered to patients in Phase 3


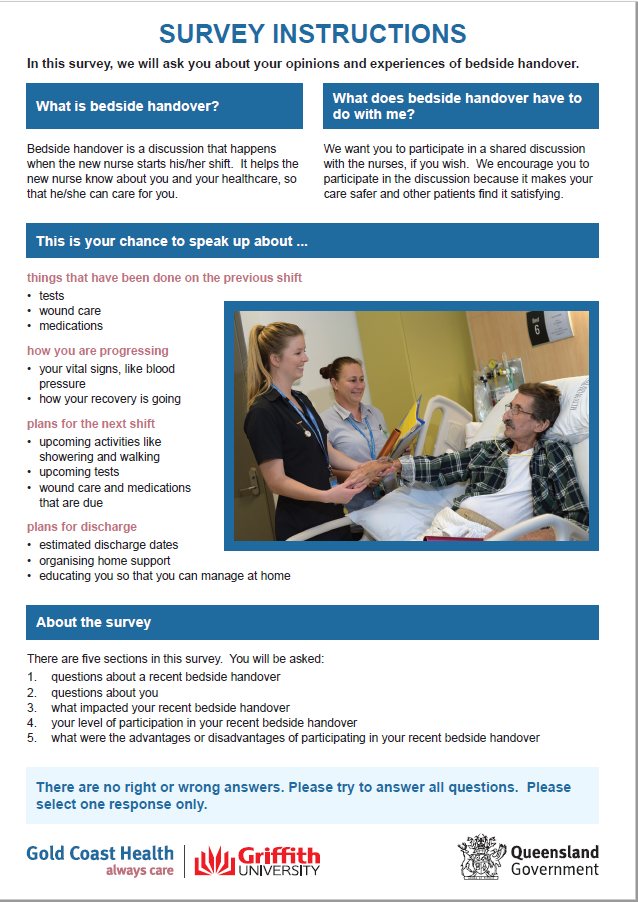


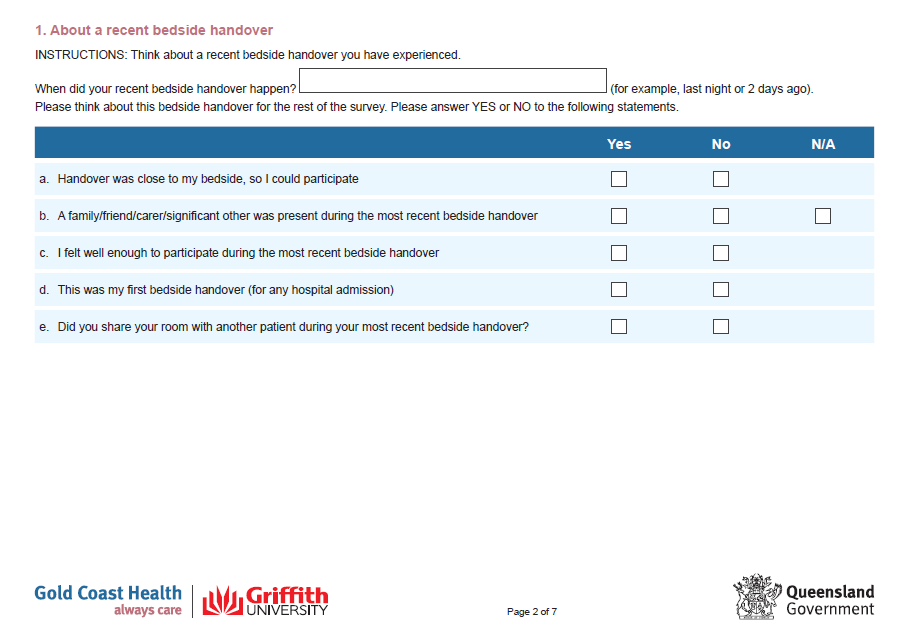


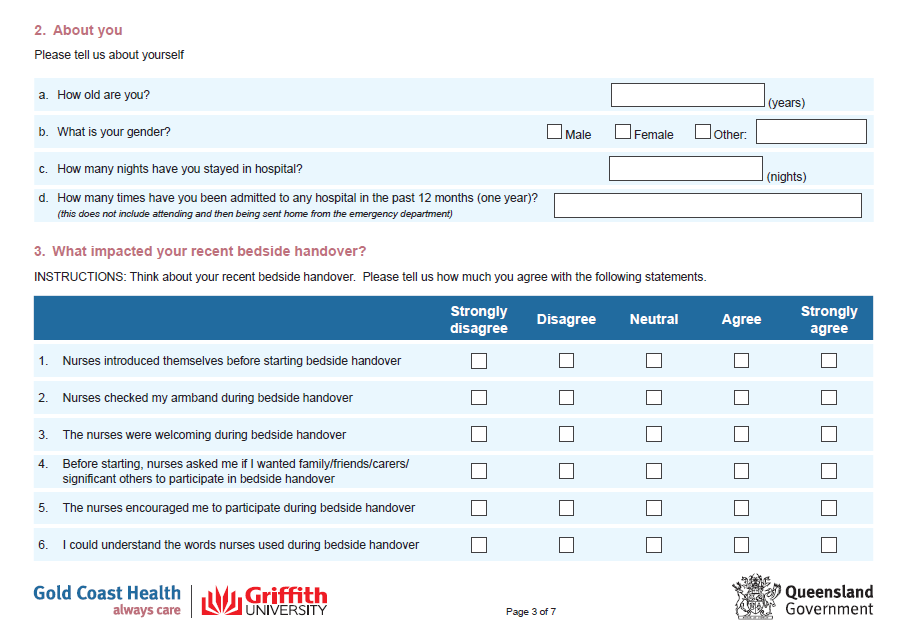


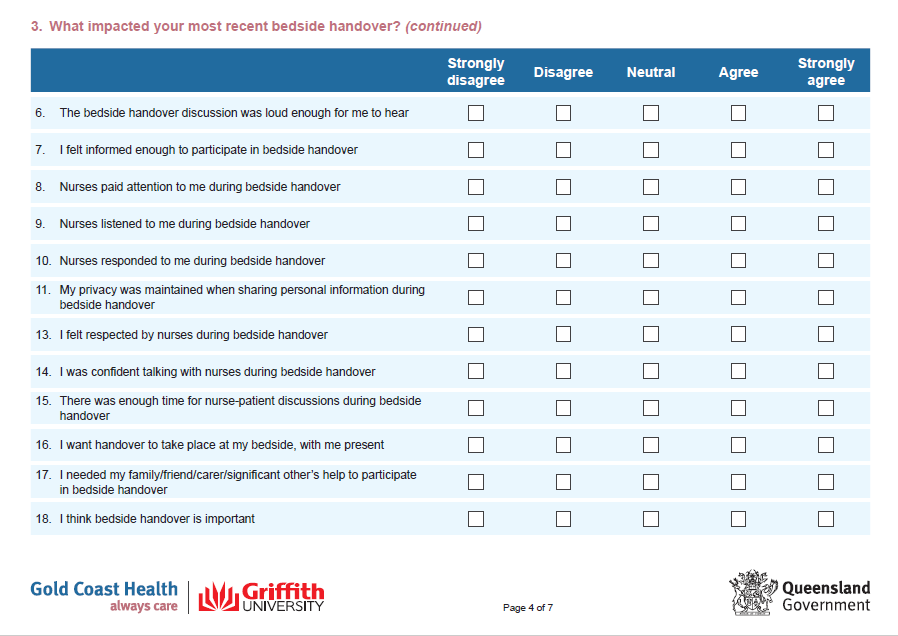


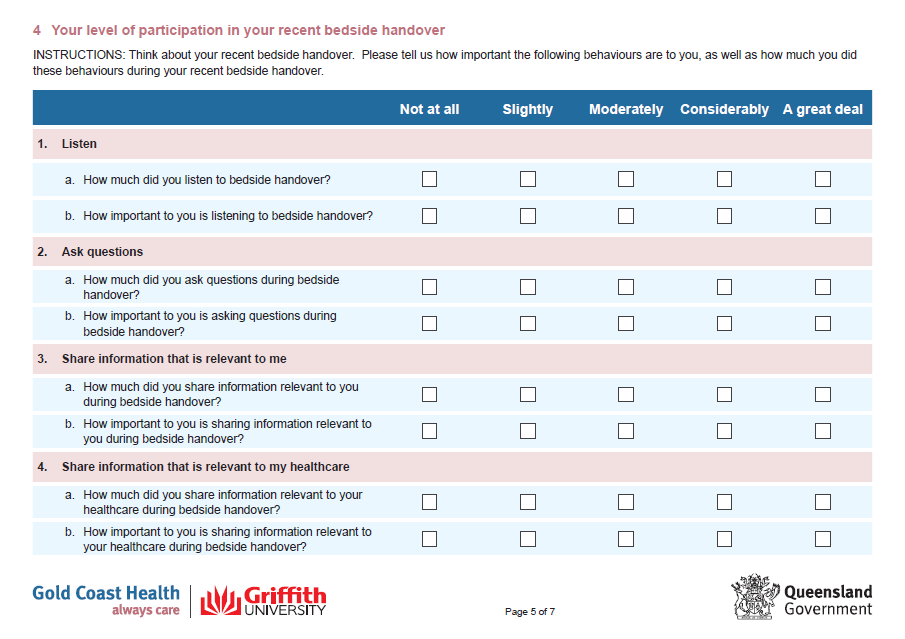


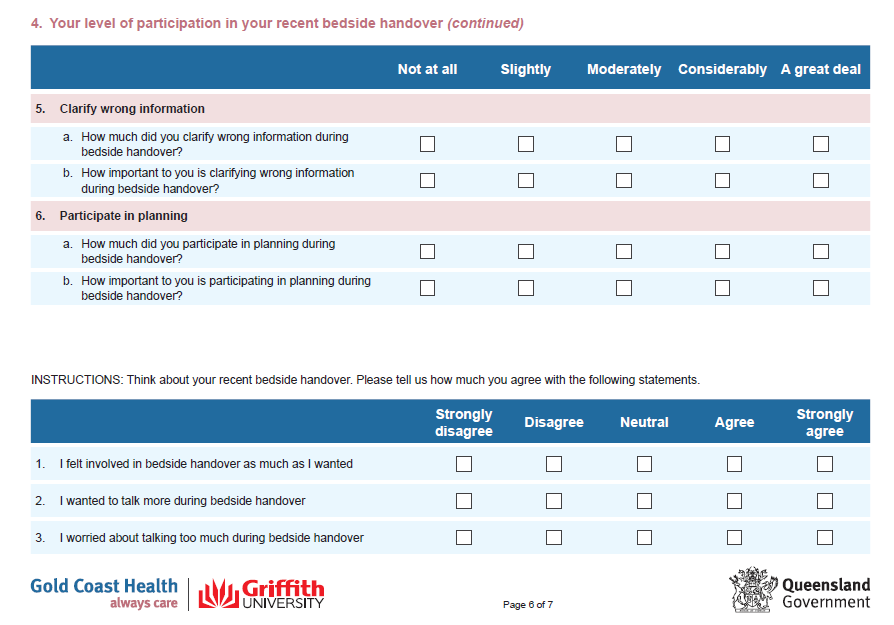


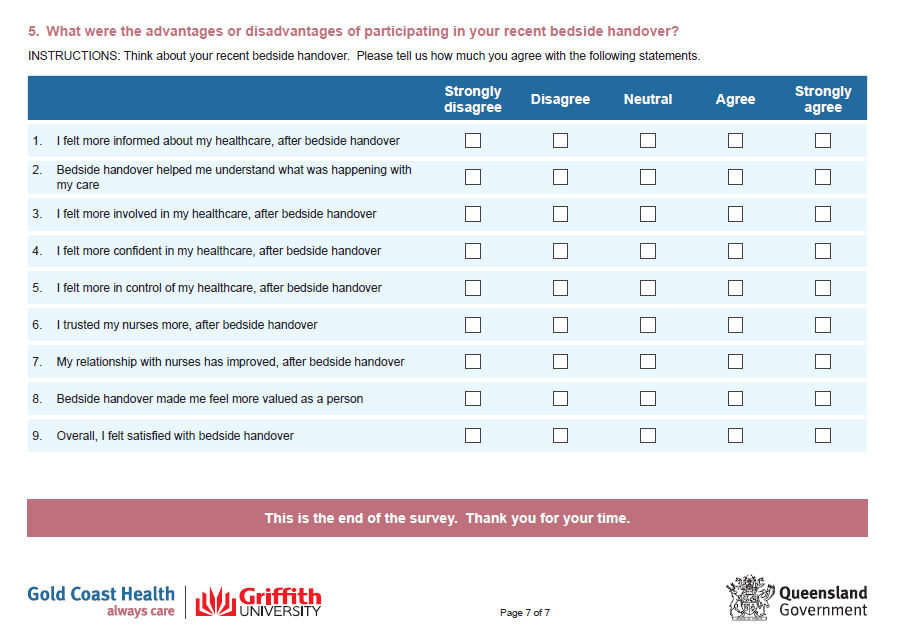


**Supplementary File 5:** Data analysis

The FACTOR program and Mplus (v8) packages were used for all analyses. To explore the internal structural validity of the 42 items, exploratory factor analyses (EFA) were conducted. EFA was appropriate since the instrument is new and its dimensional structure and therefore, optimal scoring approach is unknown. The analyses adopted three foci: 1) evaluation of overall model fit for the factor solutions; 2) evaluation of item performance; and 3) evaluation of the performance of each extracted factor.

**Suitability of the data for factor analysis**

The data were first assessed against assumptions of univariate and multivariate normality (using skew and kurtosis estimates), sampling adequacy and suitability of items for EFA (the Kaiser Myer Okin measure) and factorability of the item correlation matrix (Bartlett’s test of sphericity). Preliminary analyses showed that the 42 items were suitable for factor analysis (KMO = .85; Bartlett’s χ^2^ = 2857.10, *df* = 861, *p* < .001). However, item distributions were not univariate normal (with skew and kurtosis > + 1.00), or multivariate normal (Mardia’s estimate = 704.40, *p* < .001). To accommodate the non-normal ordinal data, a polychoric correlation matrix was used as input for factor analyses and a Robust Unweighted Least Squares (RULS) estimation procedure was utilised, which can accommodate ordinal level data, non-normality and outliers^5, 6^. The significance of parameter estimates was also checked using bias-corrected bootstrap confidence intervals. Due to the theoretical nature of the potential underlying constructs, it is expected that these will be orthogonal and therefore, an oblique rotation approach was used (Robust Promin)^7^.

**Factor retention and model fit**

Multiple indications were used to determine the optimum number of factors to extract. Kaiser’s criterion (λ > 1.00), Schwarz’s (BIC) dimensionality text, Horn’s parallel test, and the Hull method (based on the Comparative Fit Index) informed these decisions. In addition, the percentage of variance explained by a given factor solution, model fit indices, and the interpretability (i.e., simple structure) of the final solution were taken into account. Model fit indices included overall χ^2^, relative/normed χ^2^ (i.e., χ^2^/df), RMSEA with 95% CI, CFI, TLI, and SRMR. Several rules of thumb for these fit indices have achieved widespread acceptance and are followed here. Accordingly, good fit is taken to be indicated by non-significant χ^2^, χ^2^/df < 2^8^, RMSEA < .06, CFI and TLI > .95, SRMR < .08^9^.

An intentionally large item pool was generated in order to maximise content domain coverage. Therefore, strict item retention criteria were adopted to eliminate poorly performing items and ultimately, minimise respondent burden when completing the final instrument. After extracting the most accommodating factor solution (7-factor) solution, item adequacy was appraised using multiple indicators. Items with low overall communality (*h^2^* < .30) with weak primary loadings (< .63 using the benchmark of .63 as ‘very good’)^10^ or with close cross-loading (< .15 loading difference) on a secondary factor were eliminated^8^. Because the factored item set influences the performance of each individual item, an iterative approach was taken to item deletion whereby the single worst performing item was removed at a time and the solution re-estimated until simple structure was achieved. The ultimate criterion guiding factor and item decisions will be substantive interpretability of the factor solution with consideration of content validity.

The resulting factors were assessed for their quality using the ‘Overall Reliability of fully-Informative prior Oblique N-EAP scores’ index (hereon ORION), which is an assessment of the reliability of the factor scores. ORION values > 0.80 indicate precision of the factor score estimates^11^. The Factor Determinacy Index (hereon FDI) was also calculated to evaluate the accuracy of the factor score estimates given that these estimates in EFA are not unique^12^. FDI values > .90 indicate that estimates are an accurate measure of “true” score responses. Finally, construct replicability was estimated using the H-Observed index. H-Observed values > .80 suggest well-defined latent variables with high probability of remaining stable across samples (compared with H-Latent, H-Observed assesses how well a factor can be identified from the observed item scores)^12^. For completeness, and given its popularity, Cronbach’s alpha was also computed and values > .80 taken to indicate ‘good’ internal consistency.

Finally, evidence for construct validity of the factors was assessed with convergent correlation coefficients between patient and nurse reports, and converging patient demographics such as age, gender, closeness of handover to bedside, and patient rating of feeling ‘well’ enough to participate.

**Supplementary File 6:** Consumer and clinician engagement

We followed the Guidance for Reporting Involvement of Patients and the Public (GRIPP2) short form when planning and reporting this research^13^. The goal of stakeholder engagement in the research project was to enhance the acceptability and future translation of the survey into hospital practice, thus, genuinely improving patient-centred approaches to care in hospital. A health consumer representative (MS) and a senior nurse (KJ) collaborated with the lead researcher (GT) throughout the project. Prior to commencing the study, the ‘Patient Engagement Quality Guidance Tool’ was completed collaboratively by the consumer, nurse and lead researcher, to plan the consumer and nurse roles as co-researchers^14^. Once this document was complete it was used to develop a memorandum of understanding, which outlined all research team member roles; and all research team members signed this document.

Specific roles the consumer and nurse participated in included: conceiving the idea for the study (KJ), deciding to apply for funding (MS), contributing to and reviewing versions of the grant proposal (KJ, MS), reviewing the research protocol (KJ, MS), developing and refining the constructs (KJ, MS), developing survey items (KJ, MS), identifying and recruiting content experts (KJ, MS), reviewing versions of the survey during development and making decisions (KJ, MS), identifying and recruiting wards (KJ), interpreting the findings (KJ, MS), publication writing (KJ, MS), identifying sources of dissemination including hospital committees and conferences (KJ, MS), and reviewing presentations before dissemination (KJ, MS).

To monitor and measure the effectiveness of the partnership a tool was used that VicHealth developed for establishing, developing and maintaining partnerships in health promotion activities^15^. The ‘The Partnership Analysis Tool’ was applied six monthly throughout the research, completed by the consumer, nurse and lead researcher. The tool comprises seven sections that seek to measure three partnership pursuits: the need for a partnership (Sections 1-3), planning and implementing activities (Sections 4-5), and the potential for future partnerships (Sections 6-7)^15^. The survey sections are provided below^15^:

1. Determining the need for the partnership
2. Choosing partners
3. Making sure the partnerships work
4. Planning collaborative action
5. Implementing collaborative action
6. Minimising the barriers to partnerships
7. Reflecting on and continuing the partnership

Each section of the ‘The Partnership Analysis Tool’ has 5 items (35 items in total), and the maximum score is 175^15^. Scoring benchmarks are provided below^15^:

- 35-84: indicates that the whole idea of a partnership should be rigorously questioned
- 85-126: indicates that that partnership is moving in the right direction, but more attention is needed for real success
- 127-175 means the partnership is based on genuine collaboration, and the challenge is to maintain the current success

The three partners produced scores between 127-175 at all timepoints, which is above the given set parameters for concern about the partnership. The only exception was timepoint 1 where the lead researcher scored 125. The averages from the ‘The Partnership Analysis Tool’ results appear below in Table 1. Although there were individual differences in scores, the fact that our overall scores on average remained ≥144 indicated that we worked collaboratively throughout. Results were summarised after each round of surveys and shared amongst the team. The team were encouraged to provide feedback on ways to improve the partnership moving forward based on the results. The lead researcher implemented these suggestions.

**Table 1.** The Partnership Analysis Tool scores

|  | Determining need for the partnership | | Choosing partners | | Making sure partnerships work | | Planning collaborative action | | Implementing collaborative action | | Minimising the barriers to partnerships | | Reflecting on and continuing the partnership | | Overall score | |
| --- | --- | --- | --- | --- | --- | --- | --- | --- | --- | --- | --- | --- | --- | --- | --- | --- |
| Team member | Median | Range | Median | Range | Median | Range | Median | Range | Median | Range | Median | Range | Median | Range | Median | Range |
| Nurse | 22 | 21-25 | 20 | 18-21 | 20 | 19-23 | 22 | 19-23 | 21 | 20-22 | 20 | 19-23 | 20 | 19-22 | 144 | 138-156 |
| Consumer | 25 | 22-25 | 25 | 24-25 | 23 | 19-25 | 22 | 18-25 | 22 | 19-24 | 23 | 18-24 | 23 | 21-25 | 165 | 142-171 |
| Leader researcher | 23 | 18-24 | 22 | 19-24 | 20 | 15-21 | 20 | 19-22 | 21 | 18-22 | 21 | 17-24 | 21 | 19-23 | 146 | 125-159 |

**Supplementary File 7:** Final 24-item survey

|  | Items | Strongly  Disagree | Disagree | Neutral | Agree | Strongly  agree |
| --- | --- | --- | --- | --- | --- | --- |
| 1 | I felt respected by nurses during bedside handover |  |  |  |  |  |
| 2 | I was confident talking with nurses during bedside handover |  |  |  |  |  |
| 3 | My privacy was maintained when sharing personal information during bedside handover |  |  |  |  |  |
| 4 | Nurses paid attention to me during bedside handover |  |  |  |  |  |
| 5 | The nurses were welcoming during bedside handover |  |  |  |  |  |
| 6 | The bedside handover discussion was loud enough for me to hear |  |  |  |  |  |
| 7 | Nurses responded to me during bedside handover |  |  |  |  |  |
| 8 | I felt informed enough to participate in bedside handover |  |  |  |  |  |
| 9 | Nurses listened to me during bedside handover |  |  |  |  |  |
| 10 | There was enough time for nurse-patient discussions during bedside handover |  |  |  |  |  |
| 11 | I could understand the words nurses used during bedside handover |  |  |  |  |  |
|  |  | Not at all | Slightly | Moderately | Considerably | A great deal |
| 12 | How much did you share information relevant to you during bedside handover? |  |  |  |  |  |
| 13 | How much did you share information relevant to your healthcare during bedside handover? |  |  |  |  |  |
| 14 | How much did you ask questions during bedside handover? |  |  |  |  |  |
| 15 | How much did you participate in planning during bedside handover? |  |  |  |  |  |
|  |  | Strongly  Disagree | Disagree | Neutral | Agree | Strongly  agree |
| 16 | I felt more in control of my healthcare, after bedside handover |  |  |  |  |  |
| 17 | I felt more involved in my healthcare, after bedside handover |  |  |  |  |  |
| 18 | I felt more confident in my healthcare, after bedside handover |  |  |  |  |  |
| 19 | Bedside handover made me feel more valued as a person |  |  |  |  |  |
| 20 | My relationship with nurses has improved, after bedside handover |  |  |  |  |  |
| 21 | I trusted my nurses more, after bedside handover |  |  |  |  |  |
| 22 | Bedside handover helped me understand what was happening with my care |  |  |  |  |  |
| 23 | I felt more informed about my healthcare, after bedside handover |  |  |  |  |  |
| 24 | Overall, I felt satisfied with bedside handover |  |  |  |  |  |

**References**

1. Fleischer S, Berg A, Zimmermann M, Wüste K, Behrens J. Nurse-patient interaction and communication: A systematic literature review. *J Public Health*. 2009;17(5):339-353. doi:10.1007/s10389-008-0238-1

2. Evans EC. Exploring the nuances of nurse-patient interaction through concept analysis: Impact on patient satisfaction. *Nurs Sci Q*. 2016;29(1):62-70. doi:10.1177/0894318415614904

3. Tobiano G, Bucknall T, Sladdin I, Whitty JA, Chaboyer W. Patient participation in nursing bedside handover: A systematic mixed-methods review. *Int J Nurs Stud*. 2018;77:243-258. doi:10.1016/j.ijnurstu.2017.10.014

4. Polit DF, Beck CT. The content validity index: Are you sure you know what's being reported? Critique and recommendations. *Res Nurs Health*. 2006;29(5):489-497. doi:10.1002/nur.20147

5. Forero CG, Maydeu-Olivares A, Gallardo-Pujol D. Factor analysis with ordinal indicators: A Monte Carlo study comparing DWLS and ULS estimation. *Struct Equ Modeling*. 2009;16(4):625-641. doi:10.1080/10705510903203573

6. Li C-H. The performance of ML, DWLS, and ULS estimation with robust corrections in structural equation models with ordinal variables. *Psychol Methods*. 2016;21(3):369-387. doi:10.1037/met0000093

7. Lorenzo-Seva U, Ferrando PJ. Robust promin: A method for diagonally weighted factor rotation. *Rev de Psicol*. 2019;25(1):99-106. doi:10.24265/liberabit.2019.v25n1.08

8. Tabachnick BG, Fidell LS. Using multivariate statistics. 5th ed. Boston: Pearson/Allyn & Bacon; 2007.

9. Hu L-t, Bentler PM. Cutoff criteria for fit indexes in covariance structure analysis: Conventional criteria versus new alternatives. *Struct Equ Modeling*. 1999;6(1):1-55. doi:10.1080/10705519909540118

10. Comrey AL, Lee HB. A first course in factor analysis. 2nd ed. New York: Academic Press; 1992.

11. Ferrando PJ, Lorenzo-Seva U. Assessing the quality and appropriateness of factor solutions and factor score estimates in exploratory item factor analysis. *Educ Psychol Meas*. 2018;78(5):762-780. doi:10.1177/0013164417719308

12. Ferrando PJ, Lorenzo-Seva U. Assessing score determinacy, measurement quality, and closeness to unidimensionality in exploratory item factor analysis. *Educ Psychol Meas*. 2017;78(5):762–780. doi:10.1177/0013164417719308

13. Staniszewska S, Brett J, Simera I, Seers K, Mockford C, Goodlad S, et al. GRIPP2 reporting checklists: Tools to improve reporting of patient and public involvement in research. *BMJ*. 2017;358:e1-e7. doi:10.1136/bmj.j3453

14. Patient Focused Medicine Organisation. *Patient engagement quality guidance tool*. Accessed October 13, 2018. <http://patientfocusedmedicine.org/peqg/patient-engagement-quality-guidance-scenario-1.pdf>

15. VicHealth. *The partnerships analysis tool*. Accessed December 21, 2021. <https://www.vichealth.vic.gov.au/media-and-resources/publications/the-partnerships-analysis-tool>
